# Supplementary material for: Is the relative age effect just a European problem? A comprehensive analysis of birth date distribution and its impact on player selection at the 2023 FIFA Women’s World Cup
Source: PLoS One. 2025 Feb 13;20(2):e0318116. doi: 10.1371/journal.pone.0318116 (PMC11824954; doi:10.1371/journal.pone.0318116)
Supplement: Attachment — Submitted filename: Rebuttal letter PLOS ONE. female soccer.docx [file pone.0318116.s002.docx]

**Dear Reviewers,**

Thank you for dedicating your time and effort to reviewing our manuscript. We have carefully addressed all your suggestions and requirements, and we hope that this revised version aligns better with the standards for publication. Your feedback has been invaluable in improving our work, and we sincerely appreciate your guidance.

Thank you once again for your support.

Best regards,

Reviewer #1: Thank you for the opportunity to review this paper. This study examines the relative age effect in female soccer players at the 2023 Women’s World Cup. Whilst this would be a good addition to the limited number of studies exploring talent identification in youth female soccer, I believe sizable changes may need to be made to the manuscript. I have outlined these below.

Introduction
• Lines 41-42: this is a nice section, but I believe it would benefit from discussing talent identification and development of female soccer in general. There are several studies that have explored various aspects of predictors and/or mediating factors such as physical skills (Datson et al., 2020; [https://doi.org/10.1080/02640414.2019.1656323](https://secure-web.cisco.com/14Lx7KGrS7POAA1l4UEkHvaQPDjblHjJ9rLqYEHh4zLVcLqYup7fCjJvNd4-m5k4z860-X_zTV1-wDHkgXyLbjiRpQHf90JIvGsC-eVsJCUuQZdDDb8hsKsKc07niNfk6d9KPAc05G6pQLyIS4X1aWO6swJdK0vw-MHe8C1qBtym71ewNUHAonXKwd1Fx-t4rncpC3lMF0YnaL3YWjhUEtU28NWDwVFg3Jp7IIE83E_DPQnNcGlj2bqbKi3LmorEQzW7e_VHidQpOnhlQKAnjlwsbRe1REgCyOyuGLAcE7s1Lg67uMuLc4MZIPcZIKWJqtrotVYZaMTFpvKs0TAwIHQ/https%3A%2F%2Fdoi.org%2F10.1080%2F02640414.2019.1656323)), technical (e.g., Harkness-Armstrong et al., 2020; [https://doi.org/10.1080/24748668.2020.1820173](https://secure-web.cisco.com/1Ot3u1enayy4C02WaXZsJBHRPhxJhbvo_6wJJ65qcu5lO5DaMZgH686ouvzDOxPynxMxoh31Qgf0gGYCcicSXuqi-3r88MwSluYImkQzxY7M29jISPXdqGuT4GveYXDHPibMybLdfDzcIO6i_iZSe-vcTjoswn_kJy4IUKBt4kb-ZHnWx_y8XpHtgMocUdPJ8zPifcUA4mi-hTzpZeegzJVRDZUMcK3pauUD6PkPWKOy7s1cvMC2UrbLJOEg0voyKMIeiZ3hAzoHxAsDpp2hlCr6SBwfiqFYo7Vnq_F9AZvt3ZrQsfSq-SzDu_2VdZapFiz_5jlExUmkV8JO7TaDCOA/https%3A%2F%2Fdoi.org%2F10.1080%2F24748668.2020.1820173)). You could also highlight the ack of literature in this area (e.g., Curran et al., 2019; Okholm Kryger et al., 2020; [https://doi.org/10.1080/24733938.2020.1868560](https://secure-web.cisco.com/16u7JOH9yM_i1ia14QnottmW5oqksnUEPENOYNLx6FceScrcJjLH3PTLio8UlfSmt_Oty6-L7WCmcDWyGkAk9JzkAOTlhajmH5JHXwUrFxrlyy-y_yKOsZ31AtcJAq4lTP3wdQPJTkcqV8J4FOo2eUF4Au1TUNxhnA7iOwKQUmmklP3sZtNU8lwfvWNsNAfXQ9UxwkFya59ik62PyMHVYrohh0ntLEASKJ55kl7vlKI7Sa4Dk3I0vF0WVK9tiglldKvR1A9_CRIFbWxviSdFnxHo_IWQ2Q_PktY1PBlVruZr2INMVZtT1m5FFvEfqoc99EeugC9E4qSpKD_9O-2519Q/https%3A%2F%2Fdoi.org%2F10.1080%2F24733938.2020.1868560)) and the recent calls for more female specific literature examining talent identification in youth female soccer (e.g., Williams, Ford, Drust, 2023; ISBN 9781003375968; Emmonds et al., 2019; [https://doi.org/10.1186/s40798-019-0224-x](https://secure-web.cisco.com/1xcbteZZQOTy3nIL6hzLV8KtIaviY-X-OsKTiyu0CuVT4ycxqiIS15J-Tn9rtI488Ql9ID0GKVF-5kaM3ucFmTuvMHFS-g92VjDhvU8lMYdxkuXJ8d8DfuloEGLyDES5ISS74BGfi6wK4E_JvFcm8Gq37ffqYkavb99sWL44NXR4l3pxqbmOpu2psFOlcDkT0b8Fy79r_4Uviv43lJYlOU38-5vbi0dAae_PTDJ1OydSF0ZXNj4-TldiA-Y7aVN9-slyqBdxr_ReK6LIvwqXeF5Is_WACjcMKlgF3q0vLw9DYMc54TZIuhLnVsJUFSXFBstI8kcH9yd7J4DFdm54F_g/https%3A%2F%2Fdoi.org%2F10.1186%2Fs40798-019-0224-x)).
• Line 75: This is nice section, but I believe that there are several studies that may compliment this section and are not included and examine international level youth and senior female soccer layers. For example, Andrew et al. 2022 ( [https://doi.org/10.3390/children9111747](https://secure-web.cisco.com/17ckjLujqQ7C59Yh0P2FjLxbEpMNlPBzpDyPS8JWPjNCu-nBn9ouZH_B-vj_WekCuvqJbxidOdFc5S_PDC44W9gXPTFEa_W9wFL2vDDf7fhHJDLEPRmG30udkZc3P6fXPoMPuwbUOaVhDk2ZaNmMde5nSYwoSSVTys7xU38CRMPTcb2FqVPafXA__XnASaXqrJsZsxZ6uvIAoKFt6lgZRCSFf4NbjVOiRjpSlaN6THLrQhODqa1QBCUywVA_gvvZJDxypAvVCE2thKyNxJ0JfZMkPC4qJZZzKekOhUNWn-T8gYfqJbplV_cQf4jE5FY44qFp9DYTnQalPK7OdEnvOaQ/https%3A%2F%2Fdoi.org%2F10.3390%2Fchildren9111747)) explored U17, U19, and Senior Women’s soccer in the European Championships. Furthermore, Finnegan et al. 2024 explored youth national team players in the United States ([https://doi.org/10.5114/biolsport.2024.136085](https://secure-web.cisco.com/1t4mKGj2U8t-iifBII8GPBRQi16do__XDd6fpy1Qrf5CfYea7Jx28_ygtcs3ocPjDXY7clRGF0uCvZWvieZ3SsIZFYFdkEOnEC4GkVtZ8jeNFmNcJvByfAFY8g3CwyzOotFZ4O_nvua4Mpqxfv1-5zz9NMnUjFzTIxH_Vha5F5WmyTNY2ZeX7BYkYY5f0xxkkJQLiKtibN1SAGeWMSUDunkZEIxpZOlyhyBimCZuQA1tyvQ7ALuvRiUPAEY_6k24T9p2goURy8qDufwZRPNDvYL_TA705L5-CJvMvcqMYtcDGdiz6tG_DC3fIwoz1CbBo4dM34GRPPc-g36PHifth3A/https%3A%2F%2Fdoi.org%2F10.5114%2Fbiolsport.2024.136085)). Morgans et al., 2024 explored youth and senior players in Wales ([https://doi.org/10.5114/jhk%2F186563](https://secure-web.cisco.com/1V8FPKNyXL2d5UyyY9h-VTUn8ylORP0uM2-BrdWyEKpg4YcWMOl94fel0HVjsYO5rhSxu8L3Lw5SMAjflXacp3Crni4v0W_jL_0Lp-ZQ1x-3AEui-pLXkOduMwqF_OYHk7VlWpX2zwXv0LeisbnTkL4ww17OoU6zrKjp19oBTg_fgws76NBMZg-_j2n1Izp1LutIVAhBmRFLaed88TYNsEpo0BsTeF82ovwPcU5TNDgApZ_-tm274r0WJdzXoTiCvZwuj0OzawT223CcG200UphwNoQo70UsXvogXVxo_GrjDfXLocyt6DVcztr2PefwtyUIbxzCq1LOoSxdpz-LRVA/https%3A%2F%2Fdoi.org%2F10.5114%2Fjhk%252F186563)), and Gotze and Hoppe 2021 explored female soccer players in Germany ( [https://doi.org/10.3389/fpsyg.2020.587023](https://secure-web.cisco.com/1V73yZUrdYlBOfCJfUYGiFccD1Cd33m_4YKD0lK2fc6L04iM5bJmQbSOKhzkO0lbNLaPWt2za73z9_dkuwOVYL126eXGbWTkw2yl_OzfOONOQ-VLKa_Hyi_zfF1Qmsu1oD7hZZd90pGqJeOJ5Ot9MQ6ClDMzC-Cz2D1i8k3KYJBB1BRB0hWW4Ofr5ecEf_6sqZPHRGMNDyCYQ2f-Uo5lMMuk-Q-jEUcnFz95s8Hx46HCITMaFDLAvmvsyZg12YwgkK5yBujMBxWbFaagPWAFZVDmbJNR3IQNFFrBaCelgDPVWx8_fQqvGJsoPaZDsXiVS1sWKe_-swIPqX4baS3SdNQ/https%3A%2F%2Fdoi.org%2F10.3389%2Ffpsyg.2020.587023)).

Thank you for your recommendations. We have included all references suggested.

Methods
• Lines 78-81: The US Soccer Federation changed their cut off dates in 2017. Therefore, many players would have been in different cut off periods and therefore RAE, which is most prevalent youth ages would have been different for some players. They may have benefitted from the old system. Was this adjusted for?

Thank you for your valorous comment. As you well said, the change in the cutoff date was decided in August 2015 and took effect on August 1, 2017. The cutoff date was shifted from August 1 to the calendar year, mainly to align it with what is practiced in most of the rest of the world.

The average age of the USA team at the 2023 World Cup was 27 years, meaning their average age was 20 when the change occurred. At that age, women do not have any advantage related to relative age, as they have reached full maturity. Therefore, all previous cutoffs, where relative age did have an effect, were based on the August 1 cutoff.

• 100-101: Would it be better here to highlight which soccer federation (e.g., EUFA; CONCACAF etc.) they represent? For example, Americas would include Brazil and Canada. Two soccer nations that do not play in the same federation and also have very stark differences in socio-economics etc. that are known to influence talent identification.

We agree with your comment. However, we did not develop an analysis based on the international federations. We have mentioned in the discussion section the differences among regions. We assume that socio-economic context could affect to the development of players, and we will consider it for future research.

• Could you clarify, is ‘Top 4’ ranked the FIFA rankings, for the WC etc? Just to be clear for the reader. It may also be worth mentioning within the introduction around RAE being linked to success, as some research has highlighted that this may be linked to success (Augste & Lames, 2011; [https://doi.org/10.1080/02640414.2011.574719](https://secure-web.cisco.com/1sfdDWkw_L4C1njTTE8ALF9XElj3nhslfqdl1AAGk45rEUbwbXLTg95NIp1O8zaP5lbeSdOW5vfoJf24ECQIg5qG-xgp-RHFSlLrleIaatL8WToqs4sitsy1XBCSEmisG9lYf2vW63Pp2xYtb9yffjri2WiSAvjFpp4nK8ppurXNjoaFtqotgVkxYGToLLQOIxFcqLp0cZm4jnb-dTFloEl8_ygR8zD4RHag0K1OcPt0UvVFWDLjPUlmVkGEguG1Q8E98_-wJw04l1qcwEk-NuSkCzvNhJtz8RugLPfKDyhgI-4Fbh66I1FzOKnkcAVpeUv2tGFTgR2OAezRvIbg8HA/https%3A%2F%2Fdoi.org%2F10.1080%2F02640414.2011.574719)), whereas other haven’t (e.g., Andrew et al., 2022).

Thank you for this comment. As you suggested, we have included the reference in the introduction.

Results
• Well-presented tables, clear and concise.
• Lines 152 (Table 3). This is interesting, but is not worth highlighting that many of these nations have more qualified coaches simply due to the population rates as well as participation numbers?

Thank you for your comment. We have clarified this aspect in the text.

• Figure 1 needs y and x axis labels etc.
• Figures 2-3 is very difficult to view and therefore needs to be better resolution?

Following your suggestion, we have added the titles to the x and y axes in figure 1. On the other hand, Figures 2 and 3 can be enlarged without loss of image quality as they are in TIFF format.

Discussion
• Lines 200-202: This is a good point, but it may also be worth noting the differences in developmental pathways of youth female soccer players. For example, in the United States, players typically do not turn professional until they are 21-23 years of age via a draft system (i.e., they are adults; Ford et al., 2020; ISBN 9781003375968). However, many players in England engaged in senior/professional female soccer at much younger ages (17-18 years of age; Andrew et al., 2024; [https://doi.org/10.1080/02640414.2024.2356434](https://secure-web.cisco.com/1RfCncGWdIKuZfFiWzYjDn1Sk5QWnnlX-NqW4btOf_5wAkRxbsvtu2BLgJI_g5UlClRl_JnUSZ8jPaQc2cJ7_4v7Gysyrnygj4Ry23HId2H24gNP5Lnbexj7rWix7iykZQ6-6l4qDo1QxtwXDA_bNSrsY_IoObMf2tYqjT_0eh_Aw8PnEo0qEMxEPoJhx4IGofWvipkJpET6mgc20qnfMB9Hu1anLQuNCC3VMEq9Yrrqjg9hxvSmnGm30zHFGtmDVypAonZKhJ4mSRpcUNoDb2wg0Y2lHnGMUmTit-8uV-qDDTPHD3j8eeneL09dZu2U0S2ekTk7rhhm_1k0sruDuoQ/https%3A%2F%2Fdoi.org%2F10.1080%2F02640414.2024.2356434)).
• Lines 212-216: Again, how many licenses per registered player may be a better discussion point? Is this possible to gather this information.
• Lines 252-254: Really good points but I think the wider implications for talent identification and development may need to be considered here.

Thank you for all your considerations. All aspects you have mentioned have been discussed, including new references.

Reviewer #2: This article explores "Relative Age Effect" (RAE)ᅳa phenomenon where athletes born earlier in the year tend to be over represented due to selection biases participate in the 2023 FIFA Women's World Cup. According athletes birth dates, the researcher aim to uncover patterns in player selection based on birth date, depending on geographical location and playing position. The study findings suggests that European athletes, especially goalkeepers and defenders, are more likely to be affected by RAE, whereas players from other regions (Africa, America, Asia, and Oceania) show no or minimal evidence of this effect. The research try to cover women's sports area, that is traditionally overlooked compared to men's sports. RAE is a interesting research area, so better selection practices could lead to more equitable representation of athletes born throughout the year, rather than skewing in favor of those born early.

The authors use a Poisson regression as method often used to predict the likelihood of an event based on an influencing factor, in this case, the birthdate of players. However, while the Poisson model is well-suited for this type of count data, it may have some limitations.

The results indicate that birth date does play a role, though it varies by region. European players, especially goalkeepers and defenders, are more likely to be born earlier in the year. This may hint at selection biases where those with early-year birthdays have a physical advantage in youth selection, which persists into professional ranks. Conversely, players from other regions don't show this pattern as strongly, suggesting their selection practices may be less influenced by birth date.

Recommendations for Improvement

1. Authors did not Analise "years of birth" (only month) and did not mention in the introduction part "constituent year effect" (CYE) witch can provide additional information for discussion.

We appreciate the reviewer’s comment regarding the inclusion of "years of birth" and the "Constituent Year Effect" (CYE) in our study. While our analysis focuses on the "Relative Age Effect" (RAE) based on the month of birth, we acknowledge that the year of birth could provide additional insights into the outcomes, as differences in maturation between years may influence relative advantages within the same age cohort. Additionally, the "Constituent Year Effect" (CYE), as described in previous literature (Cobley et al., 2009; Wattie et al., 2015), highlights how grouping individuals from different years in a single competitive age band can contribute to differences in developmental opportunities. We will address this limitation in our discussion and explore it as a potential direction for future research.

REFERENCES

Cobley, S., Baker, J., Wattie, N., & McKenna, J. (2009). Annual age-grouping and athlete development: A meta-analytical review of relative age effects in sport. *Sports Medicine, 39*(3), 235–256. <https://doi.org/10.2165/00007256-200939030-00005>
Wattie, N., Schorer, J., & Baker, J. (2015). The relative age effect in sport: A developmental systems model. *Sports Medicine, 45*(1), 83–94. <https://doi.org/10.1007/s40279-014-0248-9>

2. The methodology is mostly appropriate, but with limitations such as the choice of statistical models, lack of controls for confounding factors, and potential selection bias. Addressing these issues could improve the robustness of the findings.
3. Elaborate on why Poisson regression was chosen and consider include additional statistical tests.

Thank you for your previous comments. Poisson Regression is one of the most popular and accepted analyses for demonstrate the relative age effect. It has been carried out in previous studies as Brustio et al. (2018), Lupo et al. (2019) and Rada et al. (2018). There are other ways to analyse RAE, as Saavedra-García exposed (2018), using logistic regression additive model. We have included this reference in the future research paragraph, and we will take into account other methods for future research.

REFERENCES

Brustio, P. R., Lupo, C., Ungureanu, A. N., Frati, R., Rainoldi, A., & Boccia, G. (2018). The relative age effect is larger in Italian soccer top-level youth categories and smaller in Serie A. *PloS one*, *13*(4), e0196253.

Lupo, C., Boccia, G., Ungureanu, A. N., Frati, R., Marocco, R., & Brustio, P. R. (2019). The beginning of senior career in team sport is affected by relative age effect. *Frontiers in Psychology*, *10*, 1465.

Rađa, A., Padulo, J., Jelaska, I., Ardigò, L. P., & Fumarco, L. (2018). Relative age effect and second-tiers: No second chance for later-born players. *PloS one*, *13*(8), e0201795.

Saavedra-Garcia, M., Matabuena, M., Montero-Seoane, A., & Fernandez-Romero, J. J. (2019). A new approach to study the relative age effect with the use of additive logistic regression models: A case of study of FIFA football tournaments (1908-2012). *PloS one*, *14*(7), e0219757.

4. Comparing the players' birth dates with general population data or amateur players could provide a baseline, can help to clarify whether these trends are specific to elite football
5. Discuss other factors (e.g., socioeconomic status) that might intersect with RAE and affect the generalization of results.

Thank you for your valuable comments (5 and 6), we have included several new references in the introduction and discussion sections, comparing our results with other investigations carried out in both amateur and professional sports.

6. In the part „Further Research“ I suggest to include qualitative component or control group analysis in future research to provide additional perspectives on RAE.

Conclusions would benefit from addressing mentioned areas for a stronger, more complete analysis. This research could contribute to the growing call for fairer talent selection processes in sports, creating opportunities for players born throughout the year to succeed equally.

Thank you again for your help in review the manuscript, we have included the last aspects you mention (comment 6 and conclusions) in the final part of document.
